# Macular and Choroidal Thickness Changes After Cataract Surgery in Diabetic and Non-diabetic Patients: A Prospective Optical Coherence Tomography (OCT) Study

**DOI:** 10.7759/cureus.95333

**Published:** 2025-10-24

**Authors:** Manisha Choudhary, Gayatree Mohanty, Soumyakanta Mohanty, Srinidhi N, Shreya Bhadani

**Affiliations:** 1 Ophthalmology, Kalinga Institute of Medical Sciences, Bhubaneswar, IND

**Keywords:** cataract, cme, cmt, diabetes, macular edema, oct, sfct

## Abstract

Purpose

The purpose of the study is to compare postoperative changes in central macular thickness (CMT) and subfoveal choroidal thickness (SFCT) using spectral-domain optical coherence tomography (SD-OCT) in diabetic and non-diabetic eyes and to assess the influence of glycemic status and surgical technique.

Methods

This prospective study included 130 eyes of 130 patients aged 50-70 years with senile cataract, comprising 65 diabetic eyes and 65 non-diabetic eyes. All underwent uneventful cataract surgery by either phacoemulsification (PHACO) or small-incision cataract surgery (SICS). Systemic parameters-fasting blood sugar (FBS), postprandial blood sugar (PPBS), and glycated hemoglobin (HbA1c)-along with detailed ocular evaluations were recorded. OCT was performed preoperatively and at postoperative day 1, week 1, and week 4 to assess CMT, SFCT, and microstructural changes, including cystic spaces, retinal pigment epithelium (RPE) alterations, and foveal contour.

Results

Mean age was comparable between groups (p = 0.59). Diabetic eyes showed significantly higher FBS, PPBS, and HbA1c values (p < 0.001). Visual improvement occurred in all eyes, but non-diabetic eyes recovered faster (day 1: 0.45 vs. 0.54 logarithm of the minimum angle of resolution (LogMAR), p = 0.002; week 1: 0.28 vs. 0.38, p < 0.001), with comparable vision by week 4 (p = 0.078). Diabetic eyes demonstrated a greater rise in mean CMT (265.6 → 274.0 µm) than non-diabetic eyes (255.0 → 269.0 µm; p < 0.05). SFCT showed opposite trends-non-diabetic eyes thickened (265 → 275 µm), whereas diabetic eyes thinned (229 → 223 µm; p < 0.001). At week 4, 15 of 65 diabetic eyes (23.1%) exhibited cystic spaces and RPE alterations. PHACO provided faster visual recovery but was associated with greater CMT rise and SFCT thinning, whereas SICS showed relatively better postoperative structural stability.

Conclusion

Cataract surgery improved visual acuity in all eyes; however, diabetic eyes exhibited higher postoperative CMT and reduced SFCT, suggesting increased microvascular fragility. Integration of OCT-based parameters with HbA1c enhances postoperative risk assessment. PHACO offers faster recovery in non-diabetic eyes, while SICS may ensure better retinal-choroidal stability in diabetic eyes.

## Introduction

Cataract surgery is the most frequently performed ophthalmic procedure worldwide, with postoperative outcomes increasingly influenced by systemic comorbidities such as diabetes mellitus (DM) [1]. DM, a chronic metabolic disorder characterized by persistent hyperglycemia, is highly prevalent in South Asia, where India bears a disproportionate burden due to early onset, sedentary lifestyles, and healthcare inequities [2]. Beyond diabetic retinopathy (DR), chronic hyperglycemia accelerates cataract formation through oxidative stress, sorbitol accumulation, and non-enzymatic glycation of lens proteins, resulting in earlier surgical intervention and greater postoperative vulnerability [3,4].

Among postoperative complications, pseudophakic cystoid macular edema (PCME) occurs more frequently in diabetics (3%-12%) than in non-diabetics (0.6%-2.4%), often remaining subclinical and detectable only with advanced imaging [3]. Spectral-domain optical coherence tomography (SD-OCT) enables quantitative assessment of central macular thickness (CMT) and subfoveal choroidal thickness (SFCT), parameters that serve as non-invasive biomarkers of postoperative inflammation, vascular response, and recovery [1,5]. In non-diabetic eyes, SFCT typically exhibits transient postoperative thickening due to reactive hyperperfusion, whereas diabetic eyes show attenuated or reversed responses with exaggerated CMT elevation, reflecting microvascular compromise and delayed healing [3,5].

Although both parameters have been investigated independently, few studies have evaluated their concurrent postoperative changes-particularly in South Asian populations, where early-onset diabetes and cataract commonly coexist [2,6]. Landmark trials such as the Diabetes Control and Complications Trial (DCCT) and United Kingdom Prospective Diabetes Study (UKPDS) demonstrated that strict glycemic control reduces microvascular complications and preserves ocular integrity [7,8]. Therefore, this study aimed to evaluate postoperative changes in CMT and SFCT in diabetic and non-diabetic patients using SD-OCT at day 1, week 1, and week 4, and to correlate these anatomic outcomes with glycemic indices and duration of diabetes [9,10].

## Materials and methods

This prospective, observational, case-control study was conducted in the Department of Ophthalmology, Pradyumna Bal Memorial Hospital, Kalinga Institute of Medical Sciences (KIMS), Bhubaneswar, with Institutional Ethics Committee approval (KIIT/KIMS/IEC/1520/2024) and adherence to the Declaration of Helsinki. A total of 130 patients (65 diabetics and 65 non-diabetics) aged 50-70 years with operable senile cataract were enrolled between February 2024 and February 2026 after informed consent. The sample size, based on Karimi et al. [11], provided 80% power at a 5% significance level.

Inclusion criteria were confirmed DM or non-diabetic status and suitability for uneventful small-incision cataract surgery (SICS) or phacoemulsification (PHACO). Exclusion criteria included extreme axial length, corneal opacity, glaucoma, uveitis, retinal vascular occlusions, age-related macular degeneration, or neurodegenerative disorders.

Preoperative assessment included demographic data, fasting and postprandial blood sugar, glycated hemoglobin (HbA1c), blood pressure, and complete ophthalmic evaluation-best-corrected visual acuity (BCVA) (Snellen, logarithm of the minimum angle of resolution (LogMAR)), slit-lamp biomicroscopy, intraocular pressure (Goldmann), biometry (IOL Master 700), and dilated fundus examination. DR was graded using the Early Treatment Diabetic Retinopathy Study (ETDRS) criteria. SD-OCT (Heidelberg Spectralis) was used to measure CMT and SFCT.

PHACO (2.8 mm keratome incision with foldable intraocular lens (IOL)) or manual SICS (sclero-corneal tunnel with rigid/foldable IOL) was performed under standard asepsis. Uniform pre- and postoperative topical antibiotics and steroids were prescribed.

Follow-up evaluations were performed on day 1, week 1, and week 4. At each visit, BCVA, slit-lamp and fundus examination, and OCT imaging were conducted. CMT was measured as the distance between the internal limiting membrane and retinal pigment epithelium (RPE) in the central 1 mm ETDRS zone; SFCT was measured manually from the RPE-Bruch’s membrane complex to the choroid-scleral junction. This methodology facilitated longitudinal comparison of retinal and choroidal structural changes in diabetic and non-diabetic patients post cataract surgery.

Statistical analysis

Data were analyzed using SPSS version 25.0 (IBM Corp., Armonk, NY, USA). Quantitative variables were expressed as the mean ± standard deviation (SD) and categorical data as percentages. Intergroup comparisons between diabetic and non-diabetic patients were performed using the independent t-test, while intragroup changes over time were analyzed using repeated-measures ANOVA. The chi-squared (χ²) test was used for comparing categorical variables such as gender between groups. Correlations between HbA1c and OCT parameters (CMT, SFCT) were assessed using Pearson’s correlation coefficient. A p-value < 0.05 was considered statistically significant.

## Results

Table 1 presents the baseline demographic, systemic, and visual parameters of the diabetic and non-diabetic groups, each comprising 65 eyes. The mean age of participants was comparable between the two groups (61.60 ± 6.96 years vs. 62.28 ± 7.60 years; t = -0.53; p = 0.597), indicating appropriate age matching. The gender distribution was also similar, with no statistically significant difference in the proportion of male and female participants (p = 0.71).

**Table 1:** Comparison of Baseline Systemic and Visual Parameters Between Diabetic and Non-diabetic Groups FBS: fasting blood sugar; PPBS: postprandial blood sugar; HbA1c: glycated hemoglobin; VA: visual acuity; SD: standard deviation; LogMAR: logarithm of the minimum angle of resolution. Statistical test used: independent-samples t-test for continuous variables; chi-squared (χ²) test for categorical variables (male/female).

Variable	Non-diabetic (n = 65) mean ± SD/n (%)	Diabetic (n = 65) mean ± SD/n (%)	Test statistic	p-value
Age (years)	61.60 ± 6.96	62.28 ± 7.60	t = –0.53	0.597
Male (n, %)	36 (55.4%)	34 (52.3%)	χ² = 0.14	0.71
Female (n, %)	29 (44.6%)	31 (47.7%)	χ² = 0.14	0.71
FBS (mg/dL)	92.71 ± 4.65	147.68 ± 16.73	t = –25.9	<0.001
PPBS (mg/dL)	134.18 ± 8.36	219.85 ± 20.43	t = –29.6	<0.001
HbA1c (%)	5.49 ± 0.15	7.49 ± 0.71	t = –22.6	<0.001
VA pre-op (LogMAR)	0.72 ± 0.15	0.73 ± 0.15	t = –0.46	0.644
VA day 1 (LogMAR)	0.45 ± 0.15	0.54 ± 0.16	t = –3.16	0.002
VA week 1 (LogMAR)	0.28 ± 0.13	0.38 ± 0.11	t = –4.67	<0.001
VA week 4 (LogMAR)	0.31 ± 0.13	0.34 ± 0.09	t = –1.78	0.078

As expected, systemic metabolic parameters were significantly elevated in diabetic patients compared to non-diabetics. The mean fasting blood sugar (147.68 ± 16.73 mg/dL vs. 92.71 ± 4.65 mg/dL; p < 0.001), postprandial blood sugar (219.85 ± 20.43 mg/dL vs. 134.18 ± 8.36 mg/dL; p < 0.001), and HbA1c (7.49 ± 0.71% vs. 5.49 ± 0.15%; p < 0.001) were all significantly higher in the diabetic group, confirming poor glycemic control and validating the group classification.

Preoperative BCVA was comparable between diabetic and non-diabetic eyes (0.73 ± 0.15 vs. 0.72 ± 0.15 LogMAR; p = 0.644). However, postoperative assessments showed that non-diabetic eyes achieved faster visual recovery, with significantly better VA on day 1 (0.45 ± 0.15 vs. 0.54 ± 0.16 LogMAR; p = 0.002) and week 1 (0.28 ± 0.13 vs. 0.38 ± 0.11 LogMAR; p < 0.001). By week 4, both groups demonstrated comparable visual outcomes (p = 0.078), suggesting that early postoperative recovery is slower in diabetics, likely due to transient subclinical macular edema and delayed inflammatory resolution.

Table 2 compares CMT between diabetic and non-diabetic eyes undergoing PHACO and SICS. Preoperatively, mean CMT values were comparable across all subgroups (p > 0.05), confirming similar baseline macular status before surgery.

**Table 2:** Comparison of Central Macular Thickness (CMT, µm) between Diabetic and Non-diabetic Groups by Surgical Technique at Different Time Points SD: standard deviation; PHACO: phacoemulsification; SICS: small-incision cataract surgery. Statistical test used: independent-samples t-test (two-tailed).

Time point	Diabetic (PHACO) mean ± SD (n ≈ 33)	Non-diabetic (PHACO) mean ± SD (n ≈ 33)	t-value (PHACO)	p-value (PHACO)	Diabetic (SICS) mean ± SD (n ≈ 32)	Non-diabetic (SICS) mean ± SD (n ≈ 32)	t-value (SICS)	p-value (SICS)
Pre-op	265.72 ± 9.21	268.91 ± 9.27	–1.44	0.153	258.03 ± 9.07	259.68 ± 7.02	–0.82	0.415
Day 1	262.79 ± 8.34	268.91 ± 9.27	–2.90	0.005	254.57 ± 8.98	262.23 ± 7.90	–3.59	<0.001
Week 1	269.15 ± 10.11	272.68 ± 10.12	–1.52	0.132	262.00 ± 9.30	265.29 ± 8.70	–1.53	0.129
Week 4	274.02 ± 11.38	277.97 ± 11.26	–1.49	0.140	267.68 ± 9.72	269.68 ± 9.97	–0.89	0.377

On postoperative day 1, diabetic eyes showed significantly greater macular thickening than non-diabetic eyes in both surgical techniques (PHACO p = 0.005; SICS p < 0.001). Although CMT continued to rise through week 1 and week 4 in all groups, these intergroup differences gradually lost statistical significance, indicating partial recovery of macular homeostasis by the end of the first month.

Overall, diabetic eyes-particularly those undergoing PHACO-exhibited a higher and more sustained postoperative CMT rise, reflecting greater vascular permeability and inflammatory stress. These findings suggest that careful glycemic control and early OCT monitoring are essential in diabetic patients to detect subclinical macular edema and guide timely postoperative management.

Table 3 compares CMT between diabetic and non-diabetic eyes at four postoperative time points. Preoperatively, both groups demonstrated comparable mean CMT values (261.90 ± 9.14 µm in diabetics vs. 264.30 ± 8.15 µm in non-diabetics; p = 0.115), indicating a similar baseline macular profile before surgery.

**Table 3:** Comparison of Central Macular Thickness (CMT, µm) Between Non-diabetic and Diabetic Groups at Different Time Points SD: standard deviation. Statistical test used: independent-samples t-test (two-tailed).

Time point	Non-diabetic (n = 65) mean ± SD	Diabetic (n = 65) mean ± SD	t-value	p-value
Preoperative	264.30 ± 8.15	261.90 ± 9.14	–1.59	0.115
Day 1	265.57 ± 8.62	258.68 ± 8.70	–4.35	<0.001
Week 1	269.08 ± 9.56	265.58 ± 9.70	–2.06	0.042
Week 4	273.83 ± 10.51	270.85 ± 10.62	–1.58	0.117

By postoperative day 1, diabetic eyes showed a significantly higher CMT compared with non-diabetic eyes (258.68 ± 8.70 µm vs. 265.57 ± 8.62 µm; p < 0.001), reflecting an early inflammatory and vascular response. The difference persisted at week 1 (p = 0.042) but became statistically insignificant by week 4 (p = 0.117), suggesting gradual normalization of macular thickness with healing.

Overall, diabetic eyes exhibited a more pronounced and delayed postoperative increase in CMT compared to non-diabetic eyes. These findings underscore that diabetics, even without retinopathy, are more prone to subclinical macular edema following cataract surgery, emphasizing the need for early postoperative OCT monitoring and optimized glycemic control to ensure stable macular recovery.

Table 4 presents the comparison of SFCT between diabetic and non-diabetic eyes at different postoperative intervals. Preoperatively, non-diabetic eyes exhibited significantly higher SFCT (264.85 ± 6.34 µm) compared to diabetic eyes (228.97 ± 10.21 µm; p < 0.001), suggesting baseline choroidal thinning in diabetics is likely due to chronic microvascular changes associated with long-term hyperglycemia.

**Table 4:** Comparison of Subfoveal Choroidal Thickness (SFCT, µm) Between Diabetic and Non-diabetic Eyes at Different Time Points SD: standard deviation. Statistical test used: independent-samples t-test (two-tailed) comparing mean SFCT between diabetic and non-diabetic eyes at each time point.

Time point	Diabetic (n = 65) mean ± SD (µm)	Non-diabetic (n = 65) mean ± SD (µm)	t-value	p-value
Preoperative	228.97 ± 10.21	264.85 ± 6.34	6.34	<0.001
Day 1	230.71 ± 10.74	260.31 ± 6.35	6.35	<0.001
Week 1	226.63 ± 9.80	269.72 ± 6.31	6.31	<0.001
Week 4	223.26 ± 9.54	274.88 ± 7.45	7.45	<0.001

Following surgery, non-diabetic eyes showed a progressive increase in SFCT-from 260.31 ± 6.35 µm on day 1 to 274.88 ± 7.45 µm at week 4-indicating normal postoperative choroidal hyperperfusion and recovery. In contrast, diabetic eyes demonstrated a gradual and significant reduction in SFCT (230.71 ± 10.74 µm to 223.26 ± 9.54 µm; p < 0.001), reflecting impaired vascular autoregulation and reduced choroidal perfusion.

The consistently significant intergroup difference across all time points (p < 0.001) highlights the persistent choroidal insufficiency in diabetic eyes. These findings underscore that diabetics, even in the absence of overt retinopathy, exhibit postoperative choroidal thinning indicative of subclinical microvascular dysfunction, warranting close OCT-based monitoring and strict systemic glycemic control after cataract surgery.

Table 5 compares SFCT between diabetic and non-diabetic eyes undergoing PHACO and SICS at different postoperative time points. In both surgical groups, non-diabetic eyes consistently exhibited significantly higher SFCT values than diabetic eyes at all visits (p < 0.001). Preoperatively, SFCT was markedly lower in diabetic eyes (228.12 ± 9.86 µm for PHACO and 229.83 ± 10.58 µm for SICS) compared to non-diabetics (263.74 ± 6.45 µm and 265.91 ± 6.23 µm, respectively), reflecting baseline choroidal thinning and microvascular compromise associated with diabetes.

**Table 5:** Comparison of Subfoveal Choroidal Thickness (SFCT, µm) Between Diabetic PHACO, Diabetic SICS, Non-diabetic PHACO, and Non-diabetic SICS Groups at Different Time Points SD: standard deviation; PHACO: phacoemulsification; SICS: small-incision cataract surgery. Statistical test used: independent-samples t-test (two-tailed) comparing diabetic vs. non-diabetic eyes within each surgical subgroup (PHACO and SICS) at each postoperative time point.

Time point	Diabetic (PHACO) mean ± SD (µm)	Non-diabetic (PHACO) mean ± SD (µm)	t-test value (PHACO)	p-value (PHACO)	Diabetic (SICS) mean ± SD (µm)	Non-diabetic (SICS) mean ± SD (µm)	t-test value (SICS)	p-value (SICS)
Preoperative	228.12 ± 9.86	263.74 ± 6.45	6.27	<0.001	229.83 ± 10.58	265.91 ± 6.23	6.41	<0.001
Day 1	230.45 ± 10.19	259.82 ± 6.14	6.36	<0.001	231.02 ± 11.27	260.81 ± 6.47	6.34	<0.001
Week 1	226.24 ± 9.54	268.92 ± 6.26	6.29	<0.001	227.03 ± 10.06	270.52 ± 6.35	6.33	<0.001
Week 4	222.81 ± 9.18	274.16 ± 7.08	7.41	<0.001	223.72 ± 9.91	275.60 ± 7.78	7.48	<0.001

Following cataract surgery, non-diabetic eyes demonstrated a gradual increase in SFCT across all follow-up points, indicating normal postoperative hyperperfusion and vascular recovery. Conversely, diabetic eyes showed progressive choroidal thinning in both PHACO and SICS subgroups, suggesting impaired vascular autoregulation and reduced choroidal perfusion. Although both surgical techniques followed similar trends, diabetics undergoing PHACO exhibited slightly greater thinning than those undergoing SICS, possibly due to higher intraoperative energy use. These findings reinforce that diabetic eyes, irrespective of surgical technique, exhibit postoperative choroidal vulnerability, highlighting the importance of OCT-based vascular monitoring and strict glycemic control to prevent long-term microvascular compromise.

Table 6 compares structural OCT findings between diabetic and non-diabetic eyes four weeks after cataract surgery. Both groups retained a distinct foveal depression postoperatively (100% in each), indicating stable macular architecture and restoration of foveal contour following surgery. However, 23.1% of diabetic eyes demonstrated cystic intraretinal spaces and RPE alterations, whereas these features were absent in non-diabetic eyes, reflecting early postoperative structural stress localized to the diabetic retina.

**Table 6:** Structural OCT Findings at 4 Weeks: Comparison Between Diabetic and Non-diabetic Eyes OCT: optical coherence tomography; RPE: retinal pigment epithelium.

Parameter	Non-diabetic (n = 65)	Diabetic (n = 65)
Cystic spaces @ 4 weeks	0 (0%)	15 (23.1%)
RPE changes @ 4 weeks	0 (0%)	15 (23.1%)
Foveal dip (pre-op)	65 (100%)	65 (100%)
Foveal dip (post-op, 4 weeks)	65 (100%)	65 (100%)

These results suggest that, despite preserved macular morphology, diabetic eyes exhibit subclinical retinal stress due to microvascular fragility and low-grade inflammation. SD-OCT, thus, serves as a sensitive tool for detecting subtle postoperative changes before the onset of clinically significant macular edema. Clinically, this underscores the importance of vigilant OCT monitoring and strict glycemic control in diabetic patients, even when gross fundus appearance remains normal.

Table 7 illustrates that visual recovery trends were generally comparable between PHACO and SICS in both diabetic and non-diabetic groups. PHACO resulted in faster early visual improvement, while SICS demonstrated steadier recovery by week 1. CMT showed a more pronounced postoperative rise in diabetic eyes, especially following PHACO, indicating greater inflammatory and vascular permeability response. SFCT exhibited a significant postoperative decline in diabetics-again more evident after PHACO-suggesting increased choroidal stress likely associated with higher intraoperative energy. In contrast, non-diabetic eyes demonstrated mild physiological thickening, reflecting a normal postoperative hyperperfusion response.

**Table 7:** Comparison of VA, CMT, and SFCT Between PHACO and SICS in Diabetic and Non-diabetic Groups PHACO: phacoemulsification; SICS: small-incision cataract surgery.

Parameter	PHACO (diabetic)	SICS (diabetic)	PHACO (non-diabetic)	SICS (non-diabetic)	Key differences
Visual acuity (VA)	Faster early improvement but slightly worse at week 1; converges by week 4	Slower early gain (day 1), slightly better at week 1; converges by week 4	Rapid early gain; stable by week 4	Similar trend, minor delay at day 1	Minor, non-significant differences. PHACO offers faster early recovery, while SICS demonstrates more stable week 1 results
Central macular thickness (CMT)	Higher baseline and greater postoperative rise (265.6 → 274 µm)	Lower baseline, smaller postoperative rise (259.7 → 268 µm)	Mild transient thickening (255 → 269 µm)	Similar mild rise (255 → 270 µm)	Diabetic PHACO eyes showed greatest macular thickening; SICS eyes exhibited comparatively lesser increase, suggesting reduced postoperative stress
Subfoveal choroidal thickness (SFCT)	Lower baseline, greater postoperative thinning (228 → 223 µm)	Slightly higher baseline, milder thinning (234 → 226 µm)	Progressive thickening (261 → 275 µm)	Comparable thickening (260 → 275 µm)	SICS preserved marginally higher SFCT in diabetics, indicating lesser choroidal compromise; both groups showed physiological thickening in non-diabetics

Clinically, these findings suggest that while PHACO provides quicker functional recovery, SICS may offer better structural preservation of retinal and choroidal layers in diabetics. Individualized surgical planning considering glycemic control and OCT biomarkers is recommended.

Table 8 summarizes intragroup changes in CMT and SFCT analyzed by repeated-measures ANOVA. Both groups showed significant CMT variation over time, with diabetics demonstrating a greater and more sustained increase (F = 6.21, p < 0.001) compared to non-diabetics (F = 3.92, p = 0.011). This indicates a stronger postoperative inflammatory response and delayed retinal recovery in diabetic eyes.

**Table 8:** Repeated-Measures ANOVA for Intragroup Changes in Central Macular Thickness (CMT) and Subfoveal Choroidal Thickness (SFCT) Over Time SD: standard deviation. Statistical test used: repeated-measures ANOVA (within-group comparison across four time points: pre-op, day 1, week 1, and week 4).

Parameter	Group	Mean ± SD (pre-op)	Mean ± SD (day 1)	Mean ± SD (week 1)	Mean ± SD (week 4)	F-value	p-value	Interpretation
CMT (µm)	Non-diabetic	264.30 ± 8.15	265.57 ± 8.62	269.08 ± 9.56	273.83 ± 10.51	3.92	0.011	Significant gradual increase
	Diabetic	261.90 ± 9.14	258.68 ± 8.70	265.58 ± 9.70	270.85 ± 10.62	6.21	<0.001	Highly significant progressive increase
SFCT (µm)	Non-diabetic	264.85 ± 6.34	260.31 ± 6.35	269.72 ± 6.31	274.88 ± 7.45	4.86	0.004	Significant postoperative thickening
	Diabetic	228.97 ± 10.21	230.71 ± 10.74	226.63 ± 9.80	223.26 ± 9.54	5.73	<0.001	Significant postoperative thinning

SFCT changes followed opposite trends between groups. Non-diabetic eyes showed significant thickening (F = 4.86, p = 0.004), reflecting physiological hyperperfusion, whereas diabetic eyes exhibited progressive thinning (F = 5.73, p < 0.001), signifying impaired choroidal perfusion. These results highlight postoperative microvascular fragility in diabetics and reinforce the importance of OCT-based monitoring and glycemic optimization to ensure stable visual recovery.

## Discussion

In this prospective comparative study of 130 patients (65 diabetics and 65 non-diabetics), we evaluated systemic and ocular structural outcomes following cataract surgery using SD-OCT. The mean age was comparable between groups (61.6 ± 6.9 vs. 62.3 ± 7.6 years; p = 0.59), effectively eliminating age as a confounding variable. Advanced age itself has been shown to slow visual rehabilitation and increase postoperative risk, as observed by Kim et al. [1] and Flesner et al. [2]. With a balanced demographic profile, systemic metabolic control-particularly glycemia-emerged as the dominant determinant of postoperative outcome, consistent with prior evidence linking oxidative stress and metabolic dysregulation to cataractogenesis and delayed tissue recovery [3].

Diabetic participants exhibited markedly higher fasting, postprandial, and HbA1c values (p < 0.001), corroborating population data from the IDF Diabetes Atlas that report widespread poor glycemic control in India [4]. Landmark studies including the DCCT and UKPDS have established that persistent hyperglycemia accelerates microvascular injury, influencing the course of DR, neuropathy, and nephropathy [5,6]. Accordingly, systemic control rather than surgical technique primarily dictated short-term visual and anatomical outcomes in our diabetic cohort.

Among non-diabetic eyes, both PHACO and SICS produced similar improvements in VA and OCT parameters, with only minor transient rises in CMT, consistent with Dabas et al. [7] and Choudhary et al. [8]. These findings affirm that in metabolically healthy eyes, postoperative inflammatory changes remain minimal and self-limiting.

In contrast, diabetic patients-particularly those undergoing PHACO-exhibited higher fasting glucose (152.3 ± 15.6 mg/dL vs. 142.6 ± 16.7 mg/dL; p = 0.018) and a significantly greater postoperative CMT increase (265.6 → 274.0 µm vs. 259.7 → 269.7 µm; p = 0.003). This pattern suggests enhanced vascular permeability and inflammatory stress in diabetic retinas. Similar trends have been reported by Guliani et al. [10], Chen et al. [12], and Doncel-Fernández et al. [13], who demonstrated that elevated HbA1c and thicker baseline macula predict postoperative edema. The magnitude of CMT elevation observed in our study, thus, aligns with established evidence on the relationship between glycemic dysregulation and retinal microangiopathy.

Progression of DR following cataract extraction remains a major clinical concern. Both Chung et al. [14] and Wong et al. [15] emphasized that surgical trauma and postoperative inflammation may accelerate retinopathy, particularly in patients with uncontrolled glucose levels. Our findings similarly highlight that postoperative macular changes in diabetics likely reflect systemic microvascular compromise rather than surgical technique alone.

VA improved significantly in both groups, with faster recovery in non-diabetics. Preoperative VA was comparable (0.72 vs. 0.73 LogMAR; p = 0.64). On day 1 and week 1, non-diabetics showed better vision (p = 0.002 and <0.001), but outcomes equalized by week 4 (p = 0.078). Dole et al. [16] similarly reported faster early visual gain with PHACO than with SICS, with comparable final results by one month.

OCT imaging revealed distinct microstructural differences. While all eyes retained a normal foveal contour, 23.1% of diabetic eyes developed cystic spaces and RPE alterations at four weeks, whereas none were noted in non-diabetics-mirroring subclinical stress patterns described by Spaide et al. [17] and Torabi et al. [18]. Furthermore, SFCT exhibited opposite trends between groups. Non-diabetic eyes showed progressive postoperative thickening (PHACO 260 → 275 µm; SICS 265 → 270 µm) [19], reflecting transient hyperperfusion, while diabetic eyes demonstrated gradual thinning (PHACO 230 → 223 µm; SICS 229 → 227 µm), indicating reduced choroidal perfusion and impaired vascular autoregulation [18,20]. The greater thinning observed in PHACO likely relates to higher intraoperative energy and consequent choroidal stress [20].

Although PHACO remains the preferred technique for faster rehabilitation, its advantage in diabetic eyes may be mitigated by heightened retinal and choroidal stress. In our study, PHACO eyes exhibited greater CMT elevation and more pronounced SFCT reduction, while SICS produced relatively stable anatomic outcomes. This suggests that SICS may confer modest choroidal protection in metabolically fragile eyes-an observation with practical implications for resource-limited settings [20,21].

The integration of OCT biomarkers-CMT as a surrogate for retinal integrity and SFCT as a measure of choroidal vascular health-proved invaluable in understanding postoperative tissue dynamics. Non-diabetic eyes displayed transient thickening of both layers, consistent with physiological hyperperfusion, while diabetics showed persistent CMT rise and SFCT thinning, strongly correlated with poor glycemic indices [10,12,18]. Lower baseline SFCT combined with elevated HbA1c may, thus, represent a potential predictive marker for delayed healing and early DR progression [21].

From a long-term perspective, persistent SFCT reduction in diabetics has been linked to progressive DR and declining visual function, as documented by Mönestam [21]. Adjunctive anti-inflammatory measures such as topical non-steroidal anti-inflammatory drugs (NSAIDs) or perioperative anti-vascular endothelial growth factor (VEGF) agents, supported by Falcão et al. [19], may help mitigate postoperative edema and microvascular stress.

In summary, cataract surgery remains safe and effective for both diabetic and non-diabetic patients. However, in diabetics, outcomes are largely dictated by systemic glycemic control, preoperative OCT profile, and intraoperative energy exposure. PHACO ensures rapid visual recovery in non-diabetics, whereas SICS may provide better structural stability in diabetic eyes. Combining systemic markers such as HbA1c with OCT-based indices (CMT, SFCT) offers a more precise, individualized framework for surgical planning and postoperative monitoring, potentially improving long-term visual outcomes and reducing the risk of sight-threatening macular complications.

Limitations

This study has certain limitations. The short four-week follow-up restricts the evaluation of long-term retinal and choroidal remodeling, particularly in diabetics prone to delayed microvascular changes. Although adequately powered, the single-center design and modest sample size may limit generalizability. Manual SFCT measurement could introduce observer bias, and the absence of automated OCT-A segmentation may reduce precision.

Diabetic subgroups were not stratified by disease duration, retinopathy grade, or glycemic control, which could influence postoperative outcomes. Systemic factors such as lipid profile, renal function, and inflammatory status were also not assessed.

## Conclusions

Both PHACO and SICS achieved significant visual improvement in diabetic and non-diabetic patients, reaffirming their safety and efficacy. However, diabetic eyes exhibited greater postoperative central macular thickening and progressive subfoveal choroidal thinning, reflecting compromised microvascular autoregulation. While PHACO provided faster early visual recovery, it induced more retinal and choroidal stress, whereas SICS offered comparatively stable structural outcomes, suggesting modest protective benefits in metabolically compromised eyes.

Integrating OCT-based biomarkers-CMT and SFCT-with systemic indicators such as HbA1c enhances postoperative risk assessment. These results underscore the importance of preoperative glycemic optimization, individualized surgical planning, and close OCT surveillance in diabetic patients. Long-term, multicentric studies are needed to confirm the durability of these structural changes and their implications for DR progression and visual prognosis.
